# The impact of arm position on image quality and radiation dose in pediatric toddler chest computed tomography: a phantom and clinical study

**DOI:** 10.3389/fmed.2026.1906166

**Published:** 2026-08-07

**Authors:** Zhen Xu, Hongrong Xu, Daisong Liu, Jiawen Zhao, Bo Liu, Jinhua Cai

**Affiliations:** Department of Radiology, Children’s Hospital of Chongqing Medical University, National Clinical Research Center for Children and Adolescents’ Health and Diseases, Ministry of Education Key Laboratory of Child Development and Disorders, Chongqing Key Laboratory of Structural Birth Defect and Reconstruction, Chongqing, China

**Keywords:** arm position, chest, computed tomography, image quality, pediatric, radiation dose

## Abstract

**Purpose:**

To investigate the effects of different arm positions on image quality and radiation dose in toddler chest computed tomography (CT).

**Methods:**

A phantom experiment and a retrospective clinical study were conducted. In the phantom study, an anthropomorphic phantom was scanned 10 times in three arm positions: arms fully elevated (Group A), arms incompletely elevated with humeri superimposed over shoulders (Group B), and arms alongside the body (Group C). In the clinical study, 120 patients aged 12–36 months were categorized into corresponding groups (Groups 1–3; *n* = 40 each). Objective image quality was assessed by CT attenuation and noise within predefined regions of interest (ROIs). Subjective quality was scored by two radiologists using a 5-point Likert scale. Radiation dose [volume computed tomography dose index and dose-length product (DLP)] was recorded.

**Results:**

Arm position significantly affected CT values and noise in the phantom study, though noise differences were modest. In patients, only one ROI showed a significant noise difference [4.30 HU, 95% confidence interval (95% CI): 0.91–7.69]. Subjective scores were highest for Group 1, lowest for Group 2, and intermediate for Group 3; all images remained diagnostically adequate. Group 2 exhibited the highest radiation dose. Although the DLP difference between Groups 1 and 3 did not reach statistical significance after correction for multiple comparisons (adjusted *p* = 0.063), the point estimate suggested a numerical trend toward lower DLP in Group 1 (Hodges–Lehmann median difference: 1.80 mGy·cm; unadjusted 95% CI: 0.53–3.63).

**Conclusion:**

For toddler chest CT, arms fully elevated is the optimal position. Arms alongside the body is an acceptable alternative when elevation is unfeasible. Humeral superimposition over the shoulders should be avoided whenever possible, as it increases radiation dose and degrades image quality.

## 1 Introduction

Medical radiation is the largest source of artificial radiation exposure for humans (1), with radiation from computed tomography (CT) examinations constituting the most significant proportion of this exposure (2–4). With societal development and the continuous installation and use of CT equipment, the number of pediatric CT examinations is rising (4, 5). Chest CT, characterized by its fast scanning speed, high density resolution, and good contrast, is widely utilized in the diagnosis and management of pediatric thoracic diseases, including those of the respiratory system, thoracic vasculature, tumors, and chest wall deformities (6, 7). Compared to adults, children are more sensitive to X-ray radiation due to their cells’ faster mitotic rates and longer expected lifespan (8). Although the absolute risks to individual patients are likely to be small, even low-dose radiation may increase the potential risk of cancer (2, 4, 8, 9). Studies have indicated that among populations undergoing CT examinations during childhood, the thyroid, lung, and breast are predicted to be the sites with the highest lifetime attributable cancer incidence due to this radiation exposure (10). Therefore, in accordance with the As Low As Reasonably Achievable (ALARA) principle, radiation dose from CT examinations should be minimized while maintaining diagnostically acceptable image quality (11).

The American Association of Physicists in Medicine states that the standard position for pediatric chest CT is with the patient in a supine position and arms above the head (12). However, achieving this standard position is not always feasible in toddlers (aged 12–36 months), as most are unable to remain still voluntarily and cooperate to complete a chest CT examination while awake. To prevent motion artifacts and subsequent rescans, uncooperative young children typically require scanning either during natural sleep or under sedation. In such scenarios, maintaining the arms fully elevated can be challenging. While existing evidence confirms that arm position affects both image quality and radiation dose in CT examinations, most relevant studies have focused on adult populations (13–16), with limited research investigating its impact specifically in toddlers. Therefore, this study aimed to investigate the effects of three different arm positions on image quality and radiation dose in pediatric toddler chest CT, using both a phantom experiment and a retrospective patient cohort analysis.

## 2 Materials and methods

### 2.1 Study design and subjects

#### 2.1.1 Phantom study

**Figure 1:**
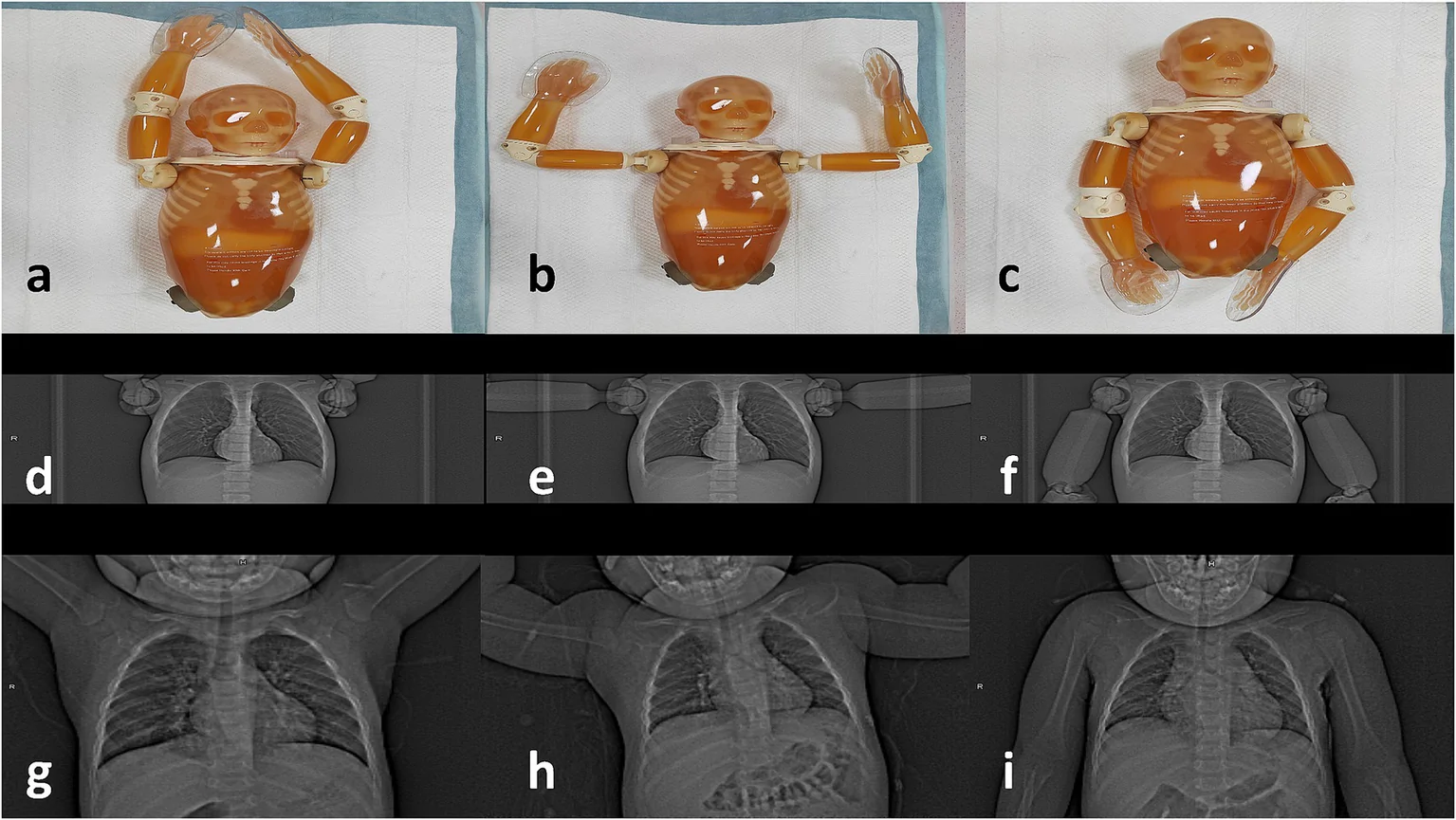
Phantom setup and corresponding CT scout images for the three arm positions. **(a–c)** Photographs of the anthropomorphic phantom in the three evaluated arm positions: **(a)** Group A (standard position), both arms fully elevated above the head; **(b)** Group B (incomplete elevation), with the humeri partially or completely superimposed over the shoulders; **(c)** Group C (arms-down position), both arms placed alongside the trunk. **(d–f)** CT scout views acquired from the phantom in the corresponding positions: **(d)** Group A, **(e)** Group B, and **(f)** Group C. **(g–i)** Representative CT scout images from the pediatric patient cohort: **(g)** Group 1, **(h)** Group 2, and **(i)** Group 3, which correspond to the arm positions illustrated in **(a)**, **(b)**, and **(c)**, respectively.

An anthropomorphic CT phantom (PBU-70; Kyoto Kagaku Corporation, Kyoto, Japan; height: 110 cm; weight: 20 kg; phantom materials: materials with an X-ray absorption rate equivalent to that of human tissues)^1^ was selected to simulate the tissues and organs from the head to the feet of a 5-year-old child (Figure 1). For this study, only the upper section of the phantom (from the head to the pelvis) was used. Three arm positions were evaluated: (A) arms elevated above the head and out of the scan range (standard clinical position); (B) arms incompletely elevated, with the humeri partially or completely superimposed over the shoulders; and (C) arms positioned alongside the body. In the phantom study, these were designated as Groups A, B, and C (Figure 1). For each arm position group, CT scanning was repeated 10 times under identical conditions in phantom study, because the anthropomorphic phantom exhibits no biological variability and each acquisition was independent, these repeated measurements were treated as independent observations for analysis. This phantom represents a 5-year-old child rather than a toddler. The phantom experiment was therefore designed to establish directional trends under controlled conditions, rather than to provide absolute values directly applicable to toddlers.

#### 2.1.2 Clinical study

The Institutional Review Board approved the retrospective study protocol. We retrospectively collected data from 120 pediatric patients aged 12–36 months who underwent unenhanced chest CT examinations. Beginning in March 2025, eligible patients were retrospectively enrolled in reverse chronological order until 40 cases were accrued for each group. Baseline characteristics, including age, sex, height, weight, and body mass index (BMI), were recorded. Children with chest wall deformities were excluded from the study. Pediatric patients were categorized into three groups (1, 2, and 3) that corresponded to the phantom arm-position groups A, B, and C, respectively (Figure 1).

### 2.2 Scanning and reconstruction parameters

All CT scans were performed on a 192-section dual-source CT scanner (Somatom Force; Siemens Healthineers, Forchheim, Germany) using a turbo flash mode protocol with Care Dose 4D fully activated. The scanning parameters were as follows: reference tube current-time product, 90 mAs; reference tube voltage, 100 kV; collimation, 192 × 0.6 mm; gantry rotation time, 0.25 s; pitch, 1.9. The scan length was set to 200 mm for the phantom. An anteroposterior scout image was acquired for positioning before the scans. The height of the examination table was adjusted to align the body’s mid-axillary line with the gantry isocenter. Image reconstruction was performed using a three-level advanced modeled iterative reconstruction (ADMIRE) algorithm with a reconstruction kernel of Br40. The source images were reconstructed as continuous axial images with a slice thickness of 1 mm.

### 2.3 Image quality evaluation

Image quality assessment was performed using a Syngo.via workstation (Siemens Healthineers, Erlangen, Germany, version VB60). In the phantom study, 14 circular regions of interest (ROIs), each with an area of 100 mm^2^, were placed across five axial anatomical levels (Supplementary Figure S1). For pediatric patient chest images, 10 ROIs were defined at three axial levels (Supplementary Figure S2). All patient ROIs were placed by a pediatric radiologist with over 10 years of experience, at three axial levels defined by consistent anatomical landmarks: (1) the upper mediastinum at the level of the third thoracic vertebra; (2) the origin of the ascending aorta; and (3) the level of the left ventricle at its maximal cross-sectional area. The size of each patient ROI was set to the largest achievable area within the target tissue, adjusted to the maximum delineable area, ranging from 75 to 100 mm^2^. All ROIs were carefully positioned to avoid confounding structures such as large vessels, airways, or focal abnormalities. The mean CT attenuation value (HU) and the corresponding standard deviation (SD) were recorded for every ROI, and the SD was used as the image noise metric. An air ROI placed in the air outside the patient’s body on the pediatric chest images served as the reference baseline, providing the background CT value and noise for the patient cohort.

Patient image series were independently reviewed by two pediatric radiologists, each with more than 10 years of experience. Image quality was assessed using a 5-point Likert scale focused on overall diagnostic suitability, taking into account noise, sharpness, and clarity of key thoracic structures (lung parenchyma, trachea, mediastinum, and chest wall). The scale ranged from 1 (non-diagnostic, severe noise/blurring) to 5 (excellent, minimal noise and optimal depiction of fine anatomy) (17). The independent scores assigned by each observer were recorded, and the average of the two scores (sum divided by two) was calculated for use in statistical analysis.

### 2.4 Radiation dose

Radiation dose metrics, including the volume computed tomography dose index (CTDIvol) and dose-length product (DLP), were recorded from the dose report sheet, with CTDIvol data specific to a 32 cm diameter dosimetry phantom.

### 2.5 Statistical analyses

Statistical analyses were performed using IBM SPSS 26 (IBM Corp., Armonk, NY, USA) and GraphPad Prism 10.1.2 (GraphPad Software, San Diego, CA, USA). Continuous variables are expressed as mean ± SD unless otherwise stated. All tests were two-sided; *p* < 0.05 was considered statistically significant.

#### 2.5.1 Phantom study

The Shapiro–Wilk test was used to assess normality of the data. The Kruskal–Wallis test was applied for group comparisons, with Dunn’s *post hoc* test and Bonferroni correction used for pairwise comparisons when the omnibus test was significant. Effect sizes are reported as *η*^2^_*H* for the omnibus test and as rank-biserial correlation (*r*) for pairwise comparisons.

#### 2.5.2 Clinical study and observer agreement

Normality was assessed using the Shapiro–Wilk test, and homogeneity of variances was assessed with Levene’s test. For data meeting both assumptions, one-way ANOVA with Tukey’s HSD *post hoc* test was applied; Welch ANOVA with Games–Howell post hoc test was used when variances were unequal. For non-normal or ordinal data, the Kruskal–Wallis test was used, followed by Dunn’s test with Bonferroni correction. Parametric effect sizes are reported as partial *η*^2^; nonparametric effect sizes as *η*^2^_*H* (omnibus) and rank-biserial correlation *r* (pairwise). Categorical variables were compared using Pearson’s *χ*^2^ test or Fisher’s exact test, with Bonferroni-adjusted pairwise comparisons when the overall test was significant; effect size is reported as Cramér’s V. Observer agreement was evaluated using weighted kappa with 95% confidence interval (95% CI) and the intraclass correlation coefficient (two-way mixed-effects model, absolute agreement, single measure).

## 3 Results

### 3.1 Objective image quality

#### 3.1.1 Phantom study

**Figure 2:**
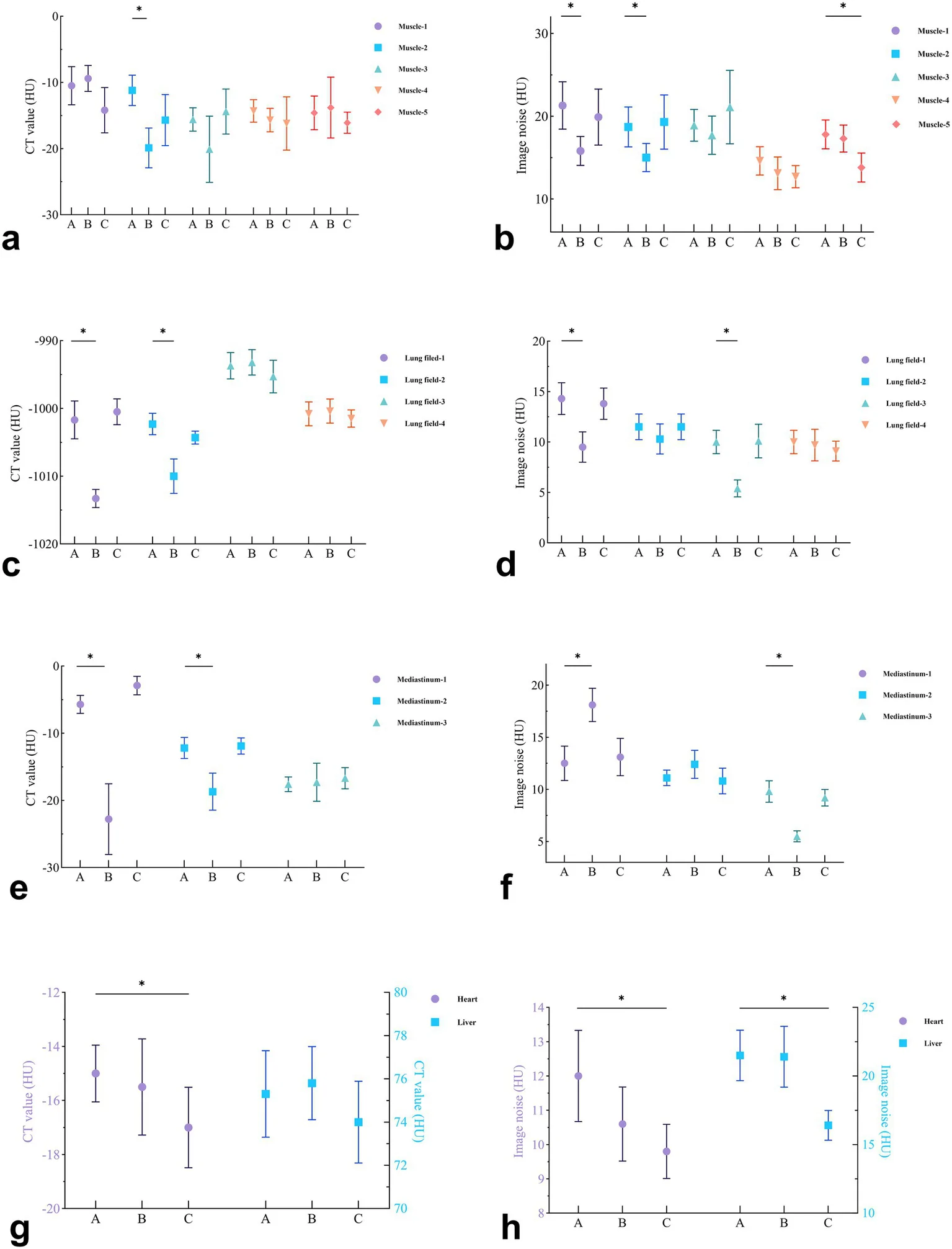
CT values and image noise of the 14 ROIs in the phantom study across the three arm position groups. Data are presented as mean and standard deviation for Groups A (arms elevated), B (incomplete elevation), and C (arms down). **(a)** CT values of the five muscle ROIs. **(b)** Noise of the muscle ROIs. **(c)** CT values of the four lung field ROIs. **(d)** Noise of the lung field ROIs. **(e)** CT values of the three mediastinum ROIs. **(f)** Noise of the mediastinum ROIs. **(g)** CT values of heart (left *y*-axis) and liver (right *y*-axis). **(h)** Noise of heart (left *y*-axis) and liver (right *y*-axis). Asterisks indicate statistically significant differences compared with Group A (*p* < 0.05). CT, computed tomography; ROI, region of interest.

In the phantom study, arm position was found to affect both CT values and image noise. Compared with Group A, Group B demonstrated statistically significant differences in CT values for 5 out of 14 ROIs, all of which showed decreased mean attenuation; these ROIs were predominantly located at the upper levels of the chest CT images. Significant differences in image noise were observed in 6 ROIs, with noise decreased in 5 ROIs and increased in 1 ROI; in these ROIs, the mean noise differences were small, on the order of approximately 5 HU. For Group C versus Group A, only 1 ROI showed a significant difference in CT values, and 3 ROIs exhibited significant differences in noise, all of which were decreased; again, the mean noise differences were within approximately 5 HU (Figure 2 and Supplementary Table S1).

#### 3.1.2 Clinical study

No statistically significant differences were found in baseline characteristics among the three patient groups (all *p* > 0.05; Table 1).

**Table 1:** Baseline characteristics of the pediatric patient cohort.

Characteristic	Group 1 (median)	Group 2 (median)	Group 3 (median)	*H*, *F* or *χ*^2^ value	*p* value	Effect size
Age (month)	24.50 ± 6.97 (26.00)	22.53 ± 7.11 (22.00)	22.60 ± 7.04 (21.00)	1.98	0.372	0.00
Sex (male/female)	22/18	24/16	28/12	1.97	0.373	0.13
Weight (kg)	11.76 ± 2.39 (11.00)	11.56 ± 1.92 (11.50)	11.35 ± 1.70 (10.93)	0.23	0.890	0.00
Height (cm)	85.47 ± 7.34 (85.50)	83.26 ± 7.50 (83.00)	83.99 ± 7.31 (85.00)	0.93	0.398	0.02
BMI	16.06 ± 2.17 (15.63)	16.69 ± 1.91 (16.69)	16.15 ± 2.01 (16.30)	3.32	0.190	0.01
Scan length (mm)	159.42 ± 14.55 (157.85)	157.60 ± 17.22 (156.80)	155.74 ± 18.06 (155.55)	0.49	0.616	0.01

BMI, body mass index; Scan length, total *z*-axis scan coverage. Negative effect size values are reported as 0.

**Figure 3:**
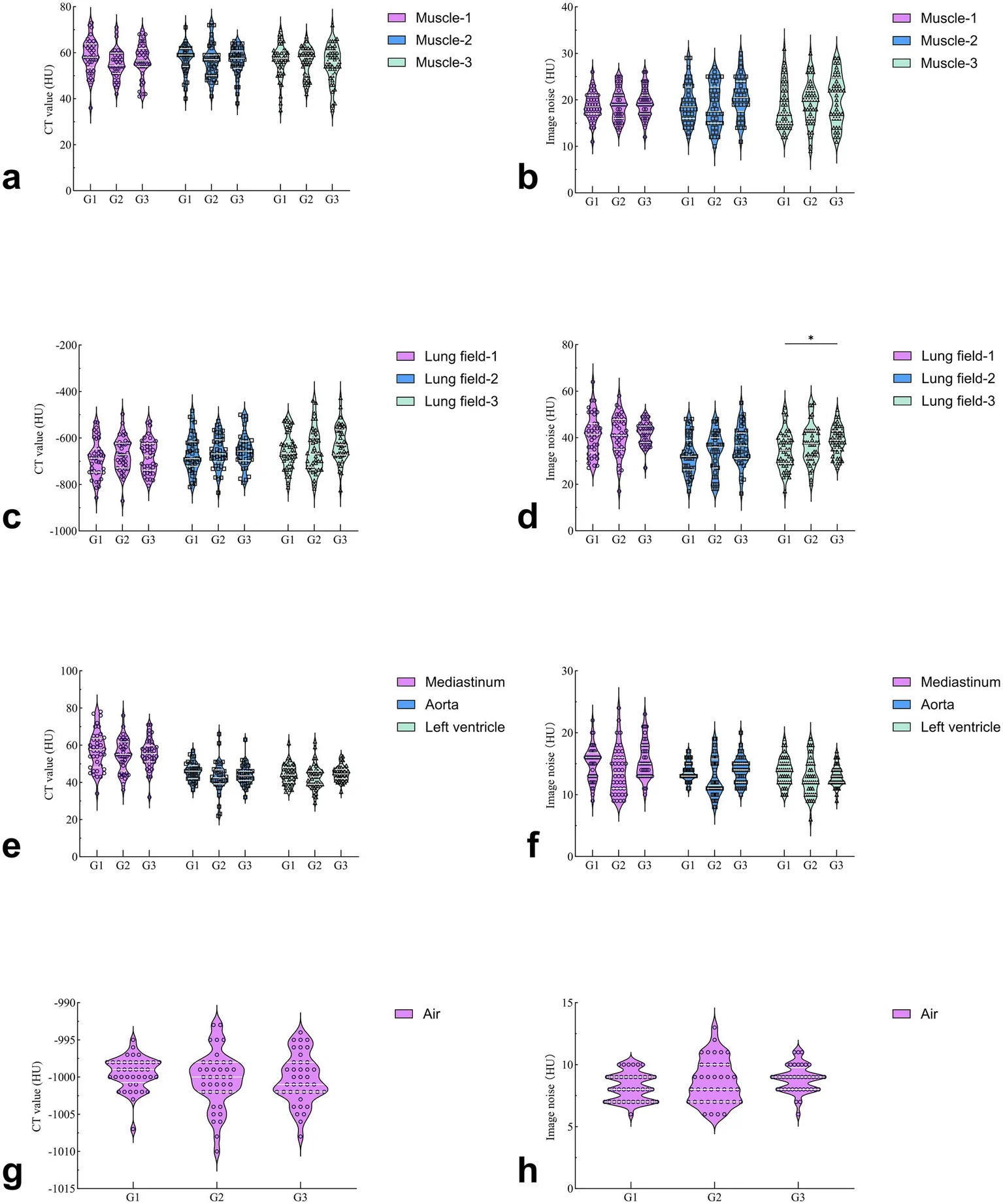
CT values and image noise of the 10 ROIs in the pediatric patient cohort across the three arm position groups. Violin plots with individual data points display the distributions for Groups 1 (arms elevated), 2 (incomplete elevation), and 3 (arms down). Within each violin, the three solid white lines denote the lower quartile, median, and upper quartile, respectively. **(a)** CT values of the three muscle ROIs. **(b)** Noise of the muscle ROIs. **(c)** CT values of the three lung field ROIs. **(d)** Noise of the lung field ROIs. **(e)** CT values of the mediastinum, aorta, and left ventricle ROIs. **(f)** Noise of the mediastinum, aorta, and left ventricle ROIs. **(g)** CT value of the air ROI. **(h)** Noise of the air ROI. Asterisks denote statistically significant differences between the indicated groups (*p* < 0.05). CT, computed tomography; ROI, region of interest.

In the pediatric cohort, no statistically significant differences were found in the baseline CT values or noise of the air ROI among the three groups. Among the remaining nine tissue ROIs, only the noise of lung field-3 showed a statistically significant pairwise difference between Group 1 and Group 3 (mean difference = 4.30 HU, 95% CI: 0.91–7.69, *p* = 0.013). For three additional ROIs, the overall Kruskal–Wallis test indicated significant differences (*p* < 0.05), but none of the *post hoc* pairwise comparisons reached significance after correction for multiple testing. No significant differences in CT values or noise were detected among the three groups for any of the other ROIs (Figure 3 and Supplementary Table S2).

### 3.2 Subjective image quality

The initial raw agreement between the two observers was 79.17% (95/120 ratings), with a weighted kappa of 0.71 (95% CI: 0.61–0.82, *Z* = 10.11, *p* < 0.01). The single-measure intraclass correlation coefficient, based on a two-way mixed-effects model with absolute agreement, was 0.79 (95% CI: 0.71–0.85, *p* < 0.01).

**Figure 4:**
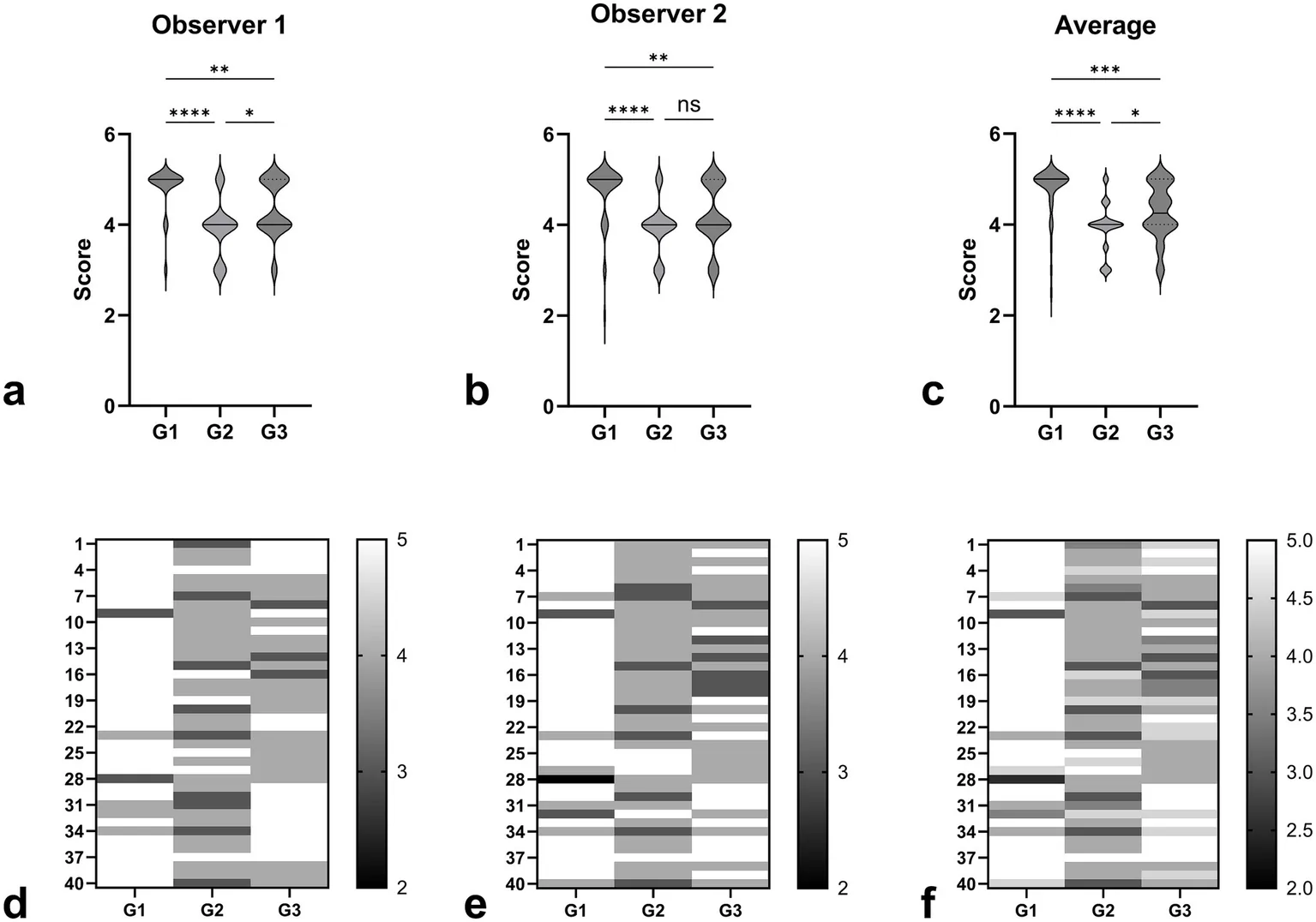
Subjective image quality scores for the three pediatric patient groups assessed by two independent observers. **(a–c)** Violin plots showing the distribution of Likert scores (1–5) for Groups 1, 2, and 3 (*n* = 40 each). Solid black lines represent medians, and dashed black lines indicate quartiles. **(a)** Scores from Observer 1. **(b)** Scores from Observer 2. **(c)** Average scores of the two observers. Asterisks denote statistically significant differences between the indicated groups: ^*^p < 0.05, ^**^p < 0.01, ^***^p < 0.001, ^****^p < 0.0001; ns, not significant. **(d–f)** Heatmaps displaying the detailed distribution of scores corresponding to **(a)**, **(b)**, and **(c)**, respectively. Grayscale intensity indicates the number of patients receiving each score, with lighter shades representing higher scores. No image was rated as 1; therefore, the scale ranges from 2 to 5. Counts are shown on the left, and the grayscale gradient is provided on the right.

For Observer 1, the image quality scores differed significantly among the three patient groups, with Group 1 receiving the highest scores, followed by Group 3, and Group 2 the lowest. Observer 2 also found significant differences among the groups, with Group 1 rated the highest; however, the difference between Group 2 and Group 3 was not statistically significant. The mean scores of the two observers showed significant differences across the three groups, again with Group 1 scoring the highest, Group 3 intermediate, and Group 2 the lowest (Figure 4 and Table 2).

**Table 2:** Subjective image quality scores of the pediatric patient cohort, presented as median (interquartile range).

Observer	Group 1	Group 2	Group 3	*H* value	*p* value	Effect size
Observer 1	5.00 (5.00–5.00)	4.00 (4.00–4.00)	4.00 (4.00–5.00)	36.57	<0.001	0.30
Observer 2	5.00 (5.00–5.00)	4.00 (4.00–4.00)	4.00 (4.00–5.00)	28.60	<0.001	0.23
Average	5.00 (5.00–5.00)	4.00 (3.63–4.00)	4.25 (4.00–5.00)	37.18	<0.001	0.30

Average, mean score of Observer 1 and Observer 2.

### 3.3 Radiation dose

**Figure 5:**
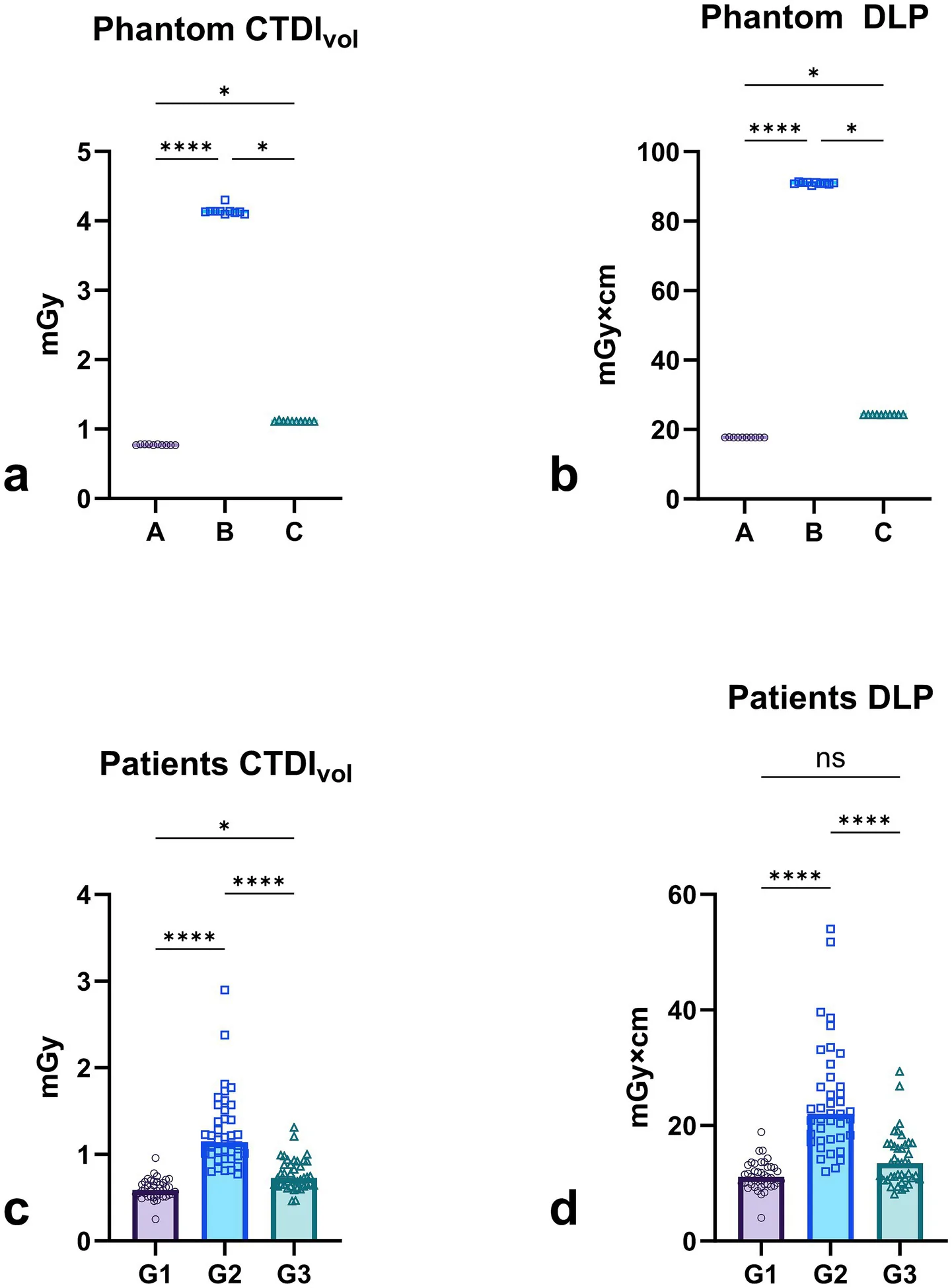
Radiation dose indices in the phantom study and pediatric patient cohort across the three arm position groups. **(a,b)** Scatter plots showing the CTDIvol and DLP for the phantom Groups A, B, and C. **(c,d)** Bar charts showing the mean values, overlaid with individual scatter points, of CTDIvol and DLP for the pediatric patient Groups 1, 2, and 3. Asterisks above the plots denote statistically significant pairwise comparisons between groups: ^*^p < 0.05, ^****^p < 0.0001; ns, not significant. CTDIvol, volume computed tomography dose index; DLP, dose-length product.

In the phantom study, both CTDIvol and DLP differed significantly among the three arm positions (Groups A, B, and C). Group B exhibited the highest dose indices, Group A the lowest, and Group C showed values intermediate between the other two groups, higher than Group A but substantially lower than Group B (Figure 5).

In the pediatric patient cohort, both CTDIvol and DLP were highest in Group 2. For CTDIvol, Group 1 had the lowest values and Group 3 was intermediate. For DLP, Group 1 and Group 3 did not differ significantly after correction for multiple comparisons (adjusted *p* = 0.063), although the point estimate suggested a numerical trend toward lower DLP in Group 1 (Hodges–Lehmann median difference: 1.80 mGy·cm; unadjusted 95% CI: 0.53–3.63). In contrast, Group 2 showed a substantially higher DLP than Group 1 (Hodges–Lehmann median difference: 10.95 mGy·cm, unadjusted 95% CI: 8.60–13.20) (Figure 5 and Supplementary Table S2).

## 4 Discussion

Chest CT is one of the most frequently performed imaging examinations in children (10). From a population perspective, even factors with seemingly minor individual effects on image quality or radiation dose may translate into a substantial cumulative impact, and therefore warrant thorough investigation (2).

In the phantom study, arm position changes led to statistically significant differences in CT values for several ROIs, likely attributable to beam-hardening artifacts caused by the arms (18, 19), which reduced CT attenuation (20, 21). This phenomenon is analogous to the beam-hardening effect observed in the posterior fossa on head CT due to interference from the skull base (20, 22). Significant differences in image noise were also observed across multiple ROIs in the phantom study, although the magnitude of change was modest, on the order of approximately 5 HU. In the patient cohort, only one ROI noise comparison reached statistical significance, and the difference was similarly small (mean difference = 4.30 HU, 95% CI: 0.91–7.69). The limited variation in image noise may be attributable to automatic exposure control (AEC), which modulates tube output based on the attenuation characteristics of the scan plane to maintain image noise at a preset level, thereby keeping noise relatively stable across acquisitions (23, 24). However, given that the human eye can discern only about 25 HU in a standard mediastinal window setting (400 HU ÷ 16 shades of gray), and even less detail in the lung window (window width ~1,500 HU, corresponding to approximately 94 HU per shade of gray), the observed noise increase of <5 HU is unlikely to be visually perceptible or clinically meaningful under either viewing condition. This is further supported by the recognition that subtle contrast differences in medical images often fall into the “subvisual” range—imperceptible even to trained radiologists—and that diagnostic performance depends critically on display settings optimized for human visual perception (25, 26).

Subjective image quality scores were highest for Group 1 (arms elevated), lowest for Group 2 (incomplete elevation), and intermediate for Group 3 (arms down). The lower scores for Groups 2 and 3, compared with Group 1, were primarily attributable to beam-hardening artifacts described above (Supplementary Figure S3) (18, 19). Nevertheless, all images were considered diagnostically adequate.

In both the phantom study and the patient cohort, intergroup comparisons revealed statistically significant differences in radiation dose, confirming that arm position indeed influences radiation exposure in pediatric chest CT. In both phantoms and patients, the arm position in which the humeri were partially or completely superimposed over the shoulders (Group B/Group 2) resulted in the highest radiation dose. Notably, the mean dose in phantom Group B was substantially higher than that in Groups A and C, whereas the dose elevation in patient Group 2 was less pronounced. A plausible explanation for this discrepancy is that, under AEC modulation, a larger effective width of the scan plane results in higher radiation output due to increased photon attenuation (24, 27–30). Fixed, substantial humeral overlap in the phantom caused a greater dose increase than the partial overlap in patients, indicating that greater overlap directly increases radiation dose. Regarding DLP, the difference between patient Groups 1 and 3 did not reach statistical significance (adjusted *p* = 0.063). This may be partly because the upper extremities of toddlers are relatively slender; placing the arms alongside the body may only minimally increase the lateral diameter of the scan plane and photon attenuation, insufficient to trigger a marked rise in AEC-driven tube output, even though CTDIvol in Group 3 was slightly higher than in Group 1. Furthermore, the mean and median scan length in Group 1 were slightly (though not significantly) greater than those in Group 3. As DLP is the product of CTDIvol and scan length, the slightly lower CTDIvol in Group 1 was partially offset by a marginally longer scan length, narrowing the DLP difference between the two groups. Nonetheless, further analysis still suggested a numerical trend toward a lower dose in Group 1 compared with Group 3 (Hodges–Lehmann median difference: 1.80 mGy·cm, unadjusted 95% CI 0.53–3.63; rank-biserial *r* = 0.26). While this dose difference is modest in absolute terms, the direction of the effect supports the preferential use of the arms-elevated position when clinically feasible.

The mean radiation dose values for all three patient groups were below the diagnostic reference levels (DRLs) recommended by the Chinese expert consensus (31), the national average from a nationwide pediatric dose survey in China (5), and the US DRLs for pediatric CT examinations (29). This may be attributable to the fact that our study was conducted at a dedicated children’s hospital using pediatric-specific CT protocols and a state-of-the-art CT scanner (5). However, individual instances of dose exceeding the DRL were observed in Group 2, indicating that this arm position carries a genuine risk of delivering unnecessarily high radiation exposure in some patients. This finding underscores the clinical importance of avoiding complete humeral superimposition, even when mean group doses remain below reference levels. Against a background of low absolute dose levels, the incremental radiation risk posed by arm position-related dose variations may be far less pronounced than the numerical increase would suggest (9, 11, 32). Nevertheless, the consistent dose elevation observed in Group 2 highlights that arm position is not merely a statistical concern but a clinically relevant determinant of radiation exposure that warrants attention in routine practice. Nonetheless, in keeping with the ALARA principle, any effort to further reduce radiation exposure while maintaining diagnostic confidence is always beneficial (33).

In routine clinical practice, toddlers often exhibit poor compliance and frequently undergo chest CT examinations while asleep, with complicating factors such as trauma, intubation, and sedation (34). Based on our findings, we propose a tiered arm-positioning strategy: (1) arms fully elevated should be used whenever feasible, providing optimal image quality and the lowest radiation dose (12, 33); (2) arms placed alongside the body is an acceptable alternative when full elevation is unattainable, offering reasonable comfort and ease of positioning with only minimal compromise in image quality and a modest dose increase; (3) arm positions involving humeral superimposition over the shoulders should be avoided whenever possible, as they substantially increase radiation dose and degrade image quality. When overlap cannot be entirely prevented, the degree of humeral superimposition should be minimized to reduce both radiation exposure and beam-hardening artifacts.

This study has several limitations. First, the phantom experiment utilized a single anthropomorphic phantom representing a 5-year-old child, whose body size did not fully match the toddler age range (12–36 months) of the patient cohort. Moreover, phantom scanning involved continuous repeated acquisitions without respiratory motion, which cannot replicate the breathing variability and subtle positional differences encountered in real patients. Nevertheless, the phantom study served as a preliminary investigation to establish the influence of arm position on image quality and radiation dose, providing a foundation for the subsequent patient study. Second, this was a retrospective analysis confined to a narrow age range, with a relatively small total sample size of 120 cases (40 per group). This may have limited the statistical power to detect certain differences, as evidenced by the borderline DLP difference between Groups 1 and 3 (adjusted *p* = 0.063), as well as the significant omnibus tests without significant pairwise comparisons for several ROIs. Additionally, the retrospective design precluded randomized assignment of arm positions and did not allow for paired intra-individual comparisons across different positions. Third, although all patient ROIs were placed by a single experienced pediatric radiologist using consistent anatomical landmarks, the ROI size had to be adjusted individually (75–100 mm^2^) to accommodate anatomical variations, which may introduce measurement variability. Nevertheless, the size range was kept as narrow as possible and all measurements were performed by the same observer to minimize inconsistency. Fourth, this investigation focused on a few representative arm positions and could not encompass the full spectrum of postures encountered in daily clinical practice. Finally, all CT images were acquired on a single scanner model using a single iterative reconstruction algorithm (ADMIRE, Siemens Healthineers). The observed dose and noise values may therefore vary on CT scanners from other vendors or with different reconstruction techniques, due to differences in AEC algorithms, image processing pipelines, and reconstruction parameters. These limitations may affect the generalizability of our findings to children of different ages and body habitus, or to examinations performed on other CT platforms. Therefore, future research would benefit from multi-center studies involving multiple scanner models, a broader range of pediatric ages and body types, a more comprehensive set of arm positions, and ideally a prospective paired design to achieve more robust and generalizable conclusions.

## 5 Conclusion

For toddler chest CT, the arms-fully-elevated position should be the first choice whenever feasible. When this position is difficult to achieve, placing the arms alongside the body represents an acceptable alternative. Arm positions involving humeral superimposition over the shoulders should be avoided whenever possible, as they increase radiation dose and degrade image quality.

## Data Availability

The raw data supporting the conclusions of this article will be made available by the authors, without undue reservation.

## Footnotes

1
www.kyotokagaku.com
