# Supplementary material for: The impact of arm position on image quality and radiation dose in pediatric toddler chest computed tomography: a phantom and clinical study
Source: Front Med (Lausanne). 2026 Aug 7;13:1906166. doi: 10.3389/fmed.2026.1906166 (PMC13493262; doi:10.3389/fmed.2026.1906166)
Supplement: Supplementary file 1 [file Supplementary_file_1.DOCX]

Supplementary Material

# 1 Supplementary Figure

**Supplementary Figure S1.** Locations of the 14 ROIs in the phantom study. (a) An anteroposterior scout image shows the five axial levels (indicated by white lines) corresponding to the measurement slices (b–f). Each axial image (b–f) displays the specific ROIs, color-coded and labeled as follows: mediastinum (pink), lung field (blue), muscle (green), heart (orange), and liver (yellow). ROI: regions of interest.

**Supplementary Figure S2**. Locations of the 10 ROIs in the pediatric patient images. (a) An anteroposterior scout image shows the three axial levels (indicated by white lines) corresponding to the measurement slices (b–d). Each axial image (b–d) displays the specific ROIs, color-coded and labeled as follows: mediastinum (pink), lung field (blue), muscle (green), air (white), aorta (orange), and left ventricle (yellow). ROI: regions of interest.

**Supplementary Figure S3.** Representative chest CT images illustrating the impact of beam-hardening artifacts across different arm positions. (a, b) Images from a patient in Group 1 (arms fully elevated) at the lung apex (a) and mid-lung (b) levels, showing no appreciable beam-hardening artifacts. (c) Image from a patient in Group 2 (incomplete arm elevation) at the lung apex level, with red arrows indicating beam-hardening artifacts in the lung apices and superior mediastinum. (d) Image from a patient in Group 3 (arms alongside the body) at the mid-lung level, with red arrows indicating beam-hardening artifacts in the dorsal aspect of the image.

# 2 Supplementary Table

**Supplementary Table S1. CT values, image noise, and radiation dose indices of the phantom group**

|  | Group A | Group B | Group C | H value | *P* value | Effect size |
| --- | --- | --- | --- | --- | --- | --- |
| Muscle-1 (CT) | -10.50±2.88 | -9.40±1.96 | -14.20±3.43 | 11.55 | 0.003 | 0.354 |
| Muscle-2 (CT) | -11.20±2.30 | -19.90±3.00 | -15.70±3.86 | 16.89 | ＜0.001 | 0.551 |
| Muscle-3 (CT) | -15.60±1.78 | -20.10±5.00 | -14.40±3.41 | 8.60 | 0.014 | 0.244 |
| Muscle-4 (CT) | -14.30±1.70 | -15.70±1.77 | -16.20±4.02 | 2.49 | 0.287 | 0.018 |
| Muscle-5 (CT) | -14.60±2.55 | -13.80±4.59 | -16.10±1.60 | 1.30 | 0.522 | 0.000 |
| Lung filed-1 (CT) | -1001.70±2.79 | -1013.30±1.34 | -1000.50±1.90 | 20.14 | ＜0.001 | 0.672 |
| Lung filed-2 (CT) | -1002.30±1.57 | -1010.00±2.54 | -1004.30±0.95 | 21.99 | ＜0.001 | 0.740 |
| Lung filed-3 (CT) | -993.70±1.95 | -993.20±1.87 | -995.30±2.41 | 4.81 | 0.090 | 0.104 |
| Lung filed-4 (CT) | -1000.80±1.75 | -1000.40±1.78 | -1001.50±1.27 | 2.40 | 0.302 | 0.015 |
| Mediastinum-1 (CT) | -5.70±1.34 | -22.80±5.27 | -2.90±1.37 | 24.40 | ＜0.001 | 0.830 |
| Mediastinum-2 (CT) | -12.20±1.32 | -18.70±2.75 | -11.90±1.20 | 19.43 | ＜0.001 | 0.646 |
| Mediastinum-3 (CT) | -17.60±1.08 | -17.30±2.83 | -16.70±1.57 | 1.75 | 0.417 | 0.000 |
| Heart (CT) | -15.00±1.05 | -15.50±1.78 | -17.00±1.49 | 7.94 | 0.019 | 0.220 |
| Liver (CT) | 75.30±2.00 | 75.80±1.69 | 74.00±1.89 | 3.74 | 0.154 | 0.064 |
| Muscle-1 (noise) | 21.30±2.87 | 15.80±1.75 | 19.90±3.38 | 14.99 | 0.001 | 0.481 |
| Muscle-2 (noise) | 18.70±2.41 | 15.00±1.70 | 19.30±3.27 | 11.48 | 0.003 | 0.351 |
| Muscle-3 (noise) | 18.90±1.91 | 17.70±2.31 | 21.10±4.43 | 5.46 | 0.065 | 0.128 |
| Muscle-4 (noise) | 14.60±1.71 | 13.10±1.97 | 12.70±1.34 | 6.20 | 0.045 | 0.156 |
| Muscle-5 (noise) | 17.80±1.75 | 17.30±1.64 | 13.80±1.75 | 15.94 | ＜0.001 | 0.516 |
| Lung filed-1 (noise) | 14.30±1.57 | 9.50±1.51 | 13.80±1.55 | 19.28 | ＜0.001 | 0.640 |
| Lung filed-2 (noise) | 11.50±1.27 | 10.30±1.49 | 11.50±1.27 | 4.37 | 0.113 | 0.088 |
| Lung filed-3 (noise) | 10.00±1.16 | 5.40±0.84 | 10.10±1.66 | 19.82 | ＜0.001 | 0.660 |
| Lung filed-4 (noise) | 10.00±1.16 | 9.70±1.57 | 9.10±0.99 | 2.53 | 0.283 | 0.020 |
| Mediastinum-1 (noise) | 12.50±1.65 | 18.10±1.60 | 13.10±1.79 | 19.66 | ＜0.001 | 0.654 |
| Mediastinum-2 (noise) | 11.10±0.74 | 12.40±1.35 | 10.80±1.23 | 8.51 | 0.014 | 0.241 |
| Mediastinum-3 (noise) | 9.80±1.03 | 5.50±0.53 | 9.20±0.79 | 20.84 | ＜0.001 | 0.698 |
| Heart (noise) | 12.00±1.33 | 10.60±1.08 | 9.80±0.79 | 12.10 | 0.002 | 0.374 |
| Liver (noise) | 21.50±1.84 | 21.40±2.22 | 16.40±1.08 | 19.31 | ＜0.001 | 0.641 |
| CTDIvol (mGy) | 0.77±0.01 | 4.14±0.06 | 1.11±0.01 | 26.64 | ＜0.001 | 0.913 |
| DLP (mGy·cm) | 17.71±0.03 | 90.92±0.34 | 24.29±0.03 | 27.28 | ＜0.001 | 0.936 |

CTDIvol, volume computed tomography dose index; DLP, dose–length product. Negative effect size values are reported as 0.

**Supplementary Table S2. CT values, image noise, and radiation dose indices of the pediatric patient cohort**

|  | Group 1 | Group 2 | Group 3 | *H* or *F* value | *P* value | Effect size |
| --- | --- | --- | --- | --- | --- | --- |
| Muscle-1 (CT) | 58.50±7.52 | 55.20±6.27 | 56.80±7.46 | 2.16 | 0.120 | 0.04 |
| Muscle-2 (CT) | 57.02±6.26 | 56.28±7.56 | 56.27±6.24 | 1.25 | 0.535 | 0.00 |
| Muscle-3 (CT) | 56.38±7.64 | 56.03±6.26 | 55.08±8.31 | 0.54 | 0.763 | 0.00 |
| Muscle-1 (noise) | 18.68±2.98 | 18.93±3.30 | 19.27±3.19 | 0.36 | 0.696 | 0.01 |
| Muscle-2 (noise) | 19.20±4.27 | 18.35±5.04 | 20.75±4.47 | 2.80 | 0.065 | 0.05 |
| Muscle-3 (noise) | 18.80±4.86 | 19.45±4.75 | 20.17±5.23 | 1.87 | 0.392 | 0.00 |
| Lung filed-1 (CT) | -689.48±81.14 | -671.87±73.08 | -677.82±72.63 | 0.56 | 0.573 | 0.01 |
| Lung filed-2 (CT) | -667.52±81.26 | -660.20±70.34 | -652.23±73.32 | 0.42 | 0.661 | 0.01 |
| Lung filed-3 (CT) | -655.70±77.95 | -655.28±92.45 | -624.30±79.08 | 1.86 | 0.160 | 0.03 |
| Lung filed-1 (noise) | 41.27±8.82 | 40.90±8.61 | 41.85±4.96 | 0.16 | 0.856 | 0.00 |
| Lung filed-2 (noise) | 32.60±8.18 | 34.03±9.78 | 36.40±8.38 | 1.90 | 0.154 | 0.03 |
| Lung filed-3 (noise) | 34.55±8.23 | 37.20±8.38 | 38.85±6.13 | 3.22 | 0.044 | 0.05 |
| Mediastinum (CT) | 57.27±10.26 | 54.63±8.87 | 55.55±7.90 | 1.56 | 0.459 | 0.00 |
| Mediastinum (noise) | 15.03±2.81 | 13.88±3.74 | 15.58±3.05 | 6.20 | 0.045 | 0.04 |
| Aorta (CT) | 45.88±4.86 | 42.68±8.25 | 44.05±5.44 | 6.09 | 0.048 | 0.04 |
| Aorta (noise) | 13.83±1.52 | 12.78±3.15 | 13.93±2.17 | 6.28 | 0.043 | 0.04 |
| Left ventricle (CT) | 44.33±5.34 | 42.85±6.77 | 44.62±4.00 | 1.20 | 0.305 | 0.02 |
| Left ventricle (noise) | 13.77±2.25 | 12.68±3.01 | 12.97±1.72 | 2.28 | 0.107 | 0.04 |
| Air (CT) | -999.25±2.84 | -1000.37±3.74 | -999.87±4.60 | 2.76 | 0.252 | 0.01 |
| Air (noise) | 8.23±1.12 | 8.60±1.78 | 8.85±1.10 | 4.74 | 0.094 | 0.02 |
| CTDIvol (mGy) | 0.60±0.11 | 1.26±0.43 | 0.77±0.18 | 77.87 | ＜0.001 | 0.65 |
| DLP (mGy·cm) | 11.33±2.53 | 24.35±9.64 | 14.04±4.61 | 63.23 | ＜0.001 | 0.52 |

CTDIvol, volume computed tomography dose index; DLP, dose–length product. Negative effect size values are reported as 0.
